# Study on a Fermented Whole Wheat: Phenolic Content, Activity on PTP1B Enzyme and In Vitro Prebiotic Properties

**DOI:** 10.3390/molecules24061120

**Published:** 2019-03-21

**Authors:** Diletta Balli, Maria Bellumori, Paolo Paoli, Giuseppe Pieraccini, Monica Di Paola, Carlotta De Filippo, Diana Di Gioia, Nadia Mulinacci, Marzia Innocenti

**Affiliations:** 1 Department of NEUROFARBA, and Multidisciplinary Centre of Research on Food Sciences (M.C.R.F.S.- Ce.R.A.), University of Florence, Via Ugo Schiff 6, 50019 Sesto F.no Firenze, Italy; diletta.balli@unifi.it (D.B.); maria.bellumori@unifi.it (M.B.); marzia.innocenti@unifi.it (M.I.); 2 Department of Experimental and Clinical Biomedical Sciences “Mario Serio”, University of Florence, Viale G.B. Morgagni 50, 50139 Firenze, Italy; paolo.paoli@unifi.it; 3 Mass Spectrometry Center (CISM), Department of Health Sciences, University of Florence, Viale G. Pieraccini 6, 50139 Firenze, Italy; giuseppe.pieraccini@unifi.it; 4 Department of Biology, University of Florence, Via Madonna del Piano, 6, Sesto Fiorentino, 50019 Firenze, Italy; monica.dipaola@unifi.it; 5 Institute of Agricultural Biology and Biotechnology, National Research Council, Via G. Moruzzi 1, 56124 Pisa, Italy; carlotta.defilippo@ibba.cnr.it; 6 Department of Agricultural and Food Sciences (DISTAL), University of Bologna, Viale Fanin 42, 40127 Bologna, Italy; diana.digioia@unibo.it

**Keywords:** cereal, schaftoside, methyl ferulate, *Lactobacillus reuteri*

## Abstract

Fermented cereals, staple foods in Asia and Africa, are recently receiving a growing interest in Western countries. The object of this work is the characterization of a fermented wheat used as a food ingredient and dietary supplement. To this aim, the phenolic composition, the activity on protein tyrosine phosphatase 1B (PTP1B), an enzyme overexpressed in type-II diabetes, the in vitro prebiotic properties on *Lactobacillus* *reuteri* and the microbial composition were investigated. Basic and acidic hydrolysis were tested for an exhaustive recovery of bound phenols: the acidic hydrolysis gave best yields. Methyl ferulate and neocarlinoside were identified for the first time in wheat. The inhibitory power of the extracts of several batches were investigated on PTP1B enzyme. The product was not able to inhibit the enzyme, otherwise, for the first time, a complete inhibition was observed for schaftoside, a major *C*-flavonoid of wheat. The microbial composition was assessed identifying *Lactobacillus, Enterococcus*, and *Pediococcus* as the main bacterial species. The fermented wheat was a suitable substrate for the grown of *L. reuteri*, recognized for its health properties in the human gut. The proposed method for phenols is easier compared to those based on strong basic hydrolysis; our results assessed the bound phenols as the major fraction, differently from that suggested by the literature for fermented cereals.

## 1. Introduction

Cereal fermentation is an ancient technique applied to improve food texture, shelf-life, digestibility, and food preservation, as well as able to remove anti-nutritional factors and toxins. It is a widely applied practice in Asian and African cultures where cereals, as staple crops, are processed before consumption. In recent years, there has been renewed interest in fermented foods in Europe, especially for the supposed health benefits linked to these foods [1]. The fermentation of cereal is reported as useful for the release as well the production of a greater amount of free phenolic compounds with respect to the unfermented substrate. This may be due to breakage of the link between bound phenols and the cell wall, but also to the induced synthesis of bioactive compounds determined by microbial communities during the fermentation process [2,3]. The main classes of phenolic compounds in cereals are benzoic derivatives, cinnamic acids, and flavonoids; they can be present in free or bound forms (esterified or etherified), linked to polysaccharides, proteins, lignin and cutin, or other cell wall constituents [4]. The literature points out that bound phenols are the main compounds responsible for the antioxidant capacity of cereals. The fermentation process, in vivo modifying the structure of the fibre constituents, leads to the formation of substrates transformed by colonic bacteria with beneficial effects on gut bacterial composition, and protective activity against several diseases and stresses [5].

A fermented product from wheat (*Triticum aestivum*), used as food ingredient and food supplement, is Lisosan^®^ G. The production is from the wholegrain, grounded, added with water and a sourdough starter, than mixed to initiate the fermentation process, the whole product is recovered after three days, and dried. Recently, Lisosan^®^ G has been object of in vivo and ex vivo studies, but few data are available on its composition. A protective activity was observed on Wistar rats fed with different dosages of Lisosan^®^ G before the use of cisplatin as nephrotoxic, neurotoxic and ototoxic agent [6]. The protective effects of Lisosan^®^ G on human microvascular endothelial cells incubated with oxidized LDL and Lisosan^®^ G were evaluated, at different concentrations of the product observing a decrease of some inflammatory markers [7]. The study of Lucchesi et al. [8] focused on the effects of Lisosan^®^ G as an antioxidant for human endothelial progenitor cells exposed to oxidative stress. These cells, treated with the product before the incubation with hydrogen peroxide, increased the cell viability and adhesion, and decreased cellular senescence. The liver was a further target of studies on Lisosan^®^ G: treatment with Lisosan^®^ G on primary cultures of rat hepatocytes in presence of hydrogen peroxide, induced the inactivation of NF-KB transcription factor involved in oxidative damage and an up-regulation of Nrf2 responsible for cytoprotection by inducing detoxifying enzymes [9]. Despite the studies focused on the evaluation of some health properties, no data concerning the phenolic content, the microbiological composition, the prebiotic effect and the activity on Protein Tyrosine Phosphatase 1B (PTP1B) enzyme are available for this wholegrain fermented product. PTP1B enzyme is recognized as an important target as negative regulator of insulin and leptin receptor signaling pathways [10]. The present work was aimed: (i) to investigate the chemical composition of Lisosan^®^ G in terms of free, bound, and total phenolic compounds, optimizing the extraction method; (ii) to investigate the ability of several batches of Lisosan^®^ G and of schaftoside to inhibit PTP1B enzyme; (iii) to assess the microbial composition of Lisosan^®^ G by sequencing of the bacterial 16 rRNA gene; and (iv) to evaluate the prebiotic effect on the growth of a common human probiotic species *Lactobacillus reuteri*, a widely distributed species recognized for its health properties in the human gut.

## 2. Results and Discussion

The phenolic molecules in the fermented wheat were studied as free, bound, and total phenols by applying different extractive procedures, some of them already suggested for wheat. The first aim was to verify if the fermentation process increases the free phenols as suggested by literature [2] diminishing the bound fraction. To better study also some minor components, Lisosan^®^ G was fractionated by semipreparative HPLC and the content of several phenolic fractions was determined by chromatographic and MS analysis. After the identification of the main phenolic constituents and the optimization of the extractive and analytical procedures, several batches were investigated. The ability of four batches of Lisosan^®^ G and schaftoside standard to inhibit the PTP1B enzyme was successively evaluated in vitro. Microbial composition was also assessed. The potential prebiotic properties of Lisosan^®^ G were tested on *L. reuteri* DSM 17938, a probiotic strain with demonstrated potential beneficial effects in treating and preventing human diseases.

### 2.1. Fractionation by Semipreparativa HPLC

**Figure 1:**
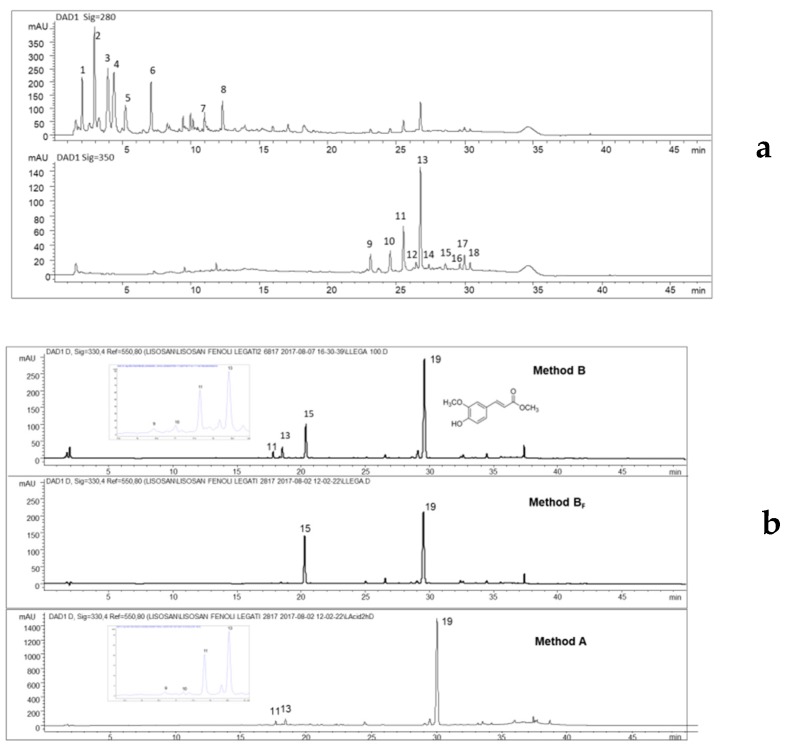
(**a**) Chromatographic profiles at 280 and 350 nm of the aqueous extract of Lisosan^®^ G on the Poroshell column, obtained applying the same gradient elution used for the semipreparative HPLC. Compounds **1**–**8**, unknowns; **9** and **10**, neocarlinoside or its isobars, isocarlinoside/carlinoside; **11**, isoschaftoside; **13**, schaftoside; **15**, ferulic acid; **16**–**18** unknowns; and (**b**) comparison of the HPLC profiles at 330 nm obtained for bound phenols with methods B and A on the whole flour, method B_F_ on the residue from free phenol extraction; **9** and **10**, neocarlinoside/isocarlinoside/carlinoside; **11**, isoschaftoside; **13**, schaftoside; **15**, ferulic acid; **19**, methyl ferulate and its chemical formula.

Regarding the free phenols, the hydroalcoholic mixture (ethanol/H_2_O 8:2, *v*/*v*) and acidic water showed comparable results, confirmed by the chromatographic profiles (data not shown). Consequently, the aqueous extract was preferred as reference sample for the semipreparative HPLC. According to Figure 1a and Table 1, some analytes detected at 280 nm (compounds **1**–**8**) were recovered; while at 350 nm *C*-flavonoids (**9**–**11** and **13**), ferulic acid (**15**), and some unknown phenols were recorded (**16**–**18**). As expected in a fermented wheat, the presence of several free amino acids was confirmed by HRMS^n^ analyses in positive ion mode, in the more polar fractions (compounds **1**–**8**), the compound **8** was identified as tryptophan (205 *m*/*z*) in co-presence with valine (118 *m*/*z*), proline (116 *m*/*z*) and leucine (132 *m*/*z*), while the other analytes (**1**–**7**) to date remained unknown. The main detected phenols were cinnamic derivatives and flavonoids, as shown in Figure 1a,b. 

**Figure 2:**
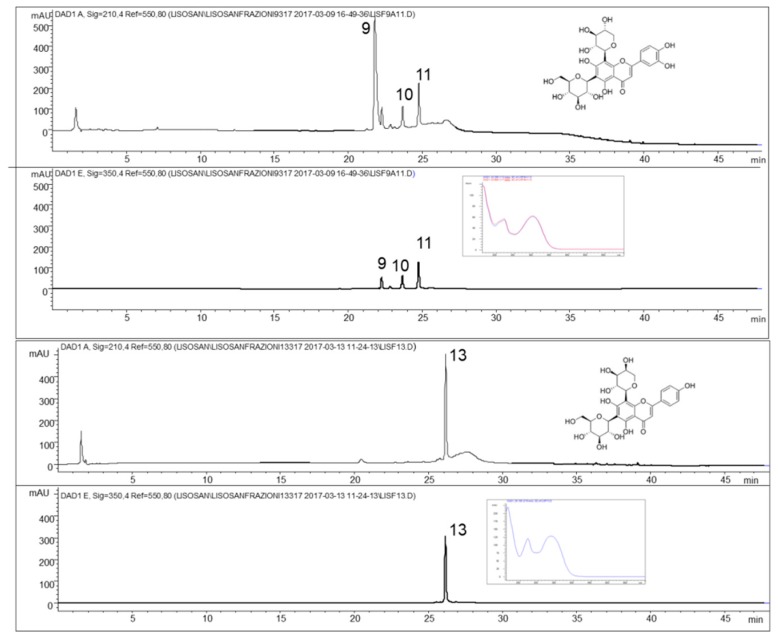
Chromatographic profiles at 210 nm and 350 nm of the two fractions containing the *C*-glycosylated flavonoids recovered by semi-preparative HPLC; **9** and **10**, neocarlinoside/isocarlinoside/carlinoside; **11**, isoschaftoside; **13**, schaftoside (UV–VIS spectra of peaks **9** and **13** and chemical formula of carlinoside and schaftoside are also shown).

From experimental data collected on the fractions by HRMS^n^ and from the results in the literature on wheat [11,12,13], it was possible to identify a group of *C*-glycosylated flavonoids (**9**–**11** and **13**) present only in two of the fractions from semipreparative HPLC (Figure 2). 

**Figure 3:**
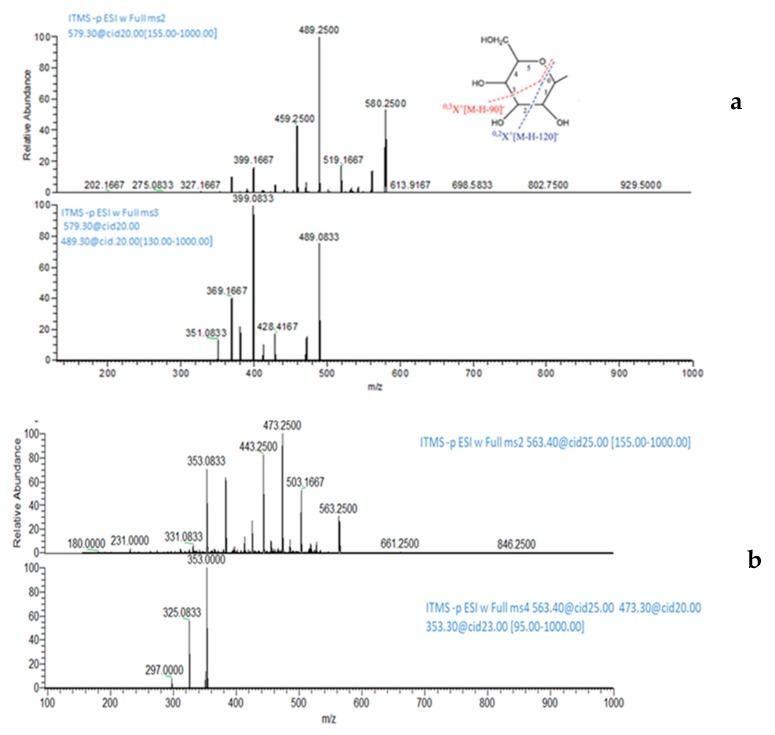
MS^2^ and MS^3^ spectra (**a**) of carlinoside/isocarlinoside/neocarlinoside (**10**) and MS^2^ and MS^4^ spectra (**b**) of isoschaftoside (**11**). All MS^n^ spectra were recorded at the optimized collision energy using wideband activation. The fragmentation of the glycosidic moiety, originating the losses of 90 and 120 mass units, is also shown.

The profiles at 210 nm, a non-selective wavelength, confirm the exclusive presence of flavonoid compounds. The mass spectra of compounds **9** and **10** (Figure 3) showed the [M − H]^−^ ion at 579 *m*/*z* in negative ionization, and similar UV–VIS spectra. The MS/MS experiments on 579 *m*/*z* showed the loss of water (561 *m*/*z*) while the two fragment ions at 489 and 459 m/z are consistent with the loss of 90 and 120 mass units, respectively. According to Iswaldi et al. [14], these losses correspond to cross-ring cleavages in the sugar moiety of *C*-glycosilated flavonoids. In light of these findings, compounds **9** and **10** were tentatively identified as carlinoside, isocarlinoside, and/or neocarlinoside. These isobaric forms have only been reported once in wheat [13], as a plant response associated to drought tolerance. Compounds **11** and **13**, with empirical formula C_26_H_28_O_14_, exhibited the same deprotonated ion at 563 *m*/*z*. The ion species at 473 *m*/*z* and 443 *m*/*z*, obtained by MS/MS dissociation of the [M − H]^−^ ion, showed again the losses of 90 and 120 mass units, respectively. From MS^3^ experiments it was possible to confirm that the common fragment ion at 353 *m*/*z* is originated from the 563 *m*/*z* (after two successive losses of 120 and 90 mass units). According to literature [15,16], it was possible to identify **11** as isoschaftoside and **13** as schaftoside.

The spectral data in positive and negative ion mode for the pool of *C*-glycosilated flavonoids are reported in Table 1. Compound **15** was identified as ferulic acid as also confirmed by the MS^2^ spectrum of its deprotonated molecular ion showing the species at 178 *m*/*z*, corresponding to the loss of a methyl group, and 134 *m*/*z* from the successive loss of carbon dioxide (59 mass units).

### 2.2. Extraction of Free, Bound, and Total Phenols

The free phenols in the first batch of Lisosan^®^ G were 38 mg/100 g (Table 2), a comparable amount to those reported by other authors for wheat, in which the concentrations were lower than 20 mg/100 g [11,17]. Regarding the bound forms, it was verified if the fermentation process can induce a release of the bound phenols. To recover this fraction, almost all the available studies on cereals reported the use of strong basic hydrolysis with NaOH (from 2 M to 10 M), usually at room temperature; the acidic hydrolysis was reported as not suitable, due to the degradation of hydroxycinnamic and benzoic acids [17,18]. Nevertheless, we observed that few data are available on the effects of different basic or acidic procedures on the chemical stability of phenols during their extraction from cereals. Consequently, with the aim of selecting the best method to effectively recover the phenolic fraction, a methanol solution with 4 M NaOH (Method B_S_) was firstly tested and compared with a softer condition with 0.1 M NaOH (Method B). HPLC-DAD analysis highlighted that the former procedure induced a partial degradation of the phenolic compounds when compared with the weaker basic hydrolysis: the use of 4 M NaOH led to the degradation of methyl ferulate converted in ferulic acid, and of compounds **9** and **10** (Table 2).

At the same time, Lisosan^®^ G was also treated according to Arranz et al. [4] to better investigate the effects of the acidic hydrolysis on the phenolic fraction. The chromatographic profiles of the sample after basic hydrolysis with 0.1 M NaOH present two main compounds: ferulic acid (**15**) and methyl ferulate (**19**), while the acidic hydrolysis (Figure 1b) shows only the presence of methyl ferulate (**19**), a compound previously detected in rice [19] but to date never reported in wheat. Presumably, the absence of methyl ferulate in literature could be attributable to the applied strong basic hydrolysis (from 2 M to 10 M) causing the formation of ferulic acid by hydrolysis of the ester bond. On the other hand, the use of a basic medium but in a weaker condition could avoid this reaction. To verify this hypothesis, pure ferulic acid was treated in the same way of Lisosan^®^ G with Method B (0.1 M NaOH in methanol); as expected, the formation of methyl ferulate was not observed and it was possible to conclude that this ester is not an artefact of the extraction process, but is naturally present in Lisosan^®^ G. Observing the profile obtained from the basic extraction (Method B_F_) of the solid residue of Lisosan^®^ G remaining after recovery of the free phenols, it was possible to assess that *C*-glycosylated flavonoids (**9**–**13**) were only present in free form (Figure 1b). In terms of extraction efficiency, the methods tested for the recovery of bound phenols gave different results. The strong basic hydrolysis (Method B_S_) led to a partial degradation of the compounds of interest (some minor *C*-flavonoids) and reduced the total phenolic recovery, in comparison to the other applied methods (Table 2a). A strong increase in solvent/dry sample ratio (from method B_F_ 1:25 *w*/*v*, to method B, 1:100 *w*/*v*) guaranteed a better recovery (up to 60% higher) in terms of total phenols (Table 2a). 

The basic hydrolysis (B) and the acidic procedure (A) applied to LG_1_ batch were the more advantageous, giving the highest yields in total phenols, with similar values of 279 mg/100 g and 265 mg/100 g, respectively. It is worth noting that the acidic hydrolysis (Table 2b, column LG_1_) maintained almost the same amount of the *C*-glycosylated flavonoids (compounds **9**–**11** and **13**) extracted with the simple procedure for free phenols (Table 2a, column FP). The acidic hydrolysis was chosen as a reliable method to compare the four Lisosan^®^ G batches for the following reasons: (i) the quantitative data from the methods A were almost the same obtained applying the basic procedure B, but in the latter case applying a four times higher extractive ratio, (ii) the phenolic compounds are chemically stable in acidic media differently to what can happen in the basic media, in which the more hydroxylated flavonoids can go toward a partial degradation, and (iii) the same acid procedure applied to other cereals (data not shown) gave again the highest recovery in terms of total phenols.

The first batch (LG_1_) was the richest in terms of total phenolic content; the LG_2_ and LG_3_ resulted very similar otherwise the LG_4_ was the poorest (Table 2b). These variable amounts of the phenolic components is in agreement with what expected for a natural product with a complex composition. Overall, in light with the findings in Table 2, it was possible to conclude that this type of fermentation is not able to increase the release of the bound phenolic fraction that remains higher than 80%, more or less the same amount measured in unfermented wheat [17].

### 2.3. Inhibition of PTP1B

**Figure 4:**
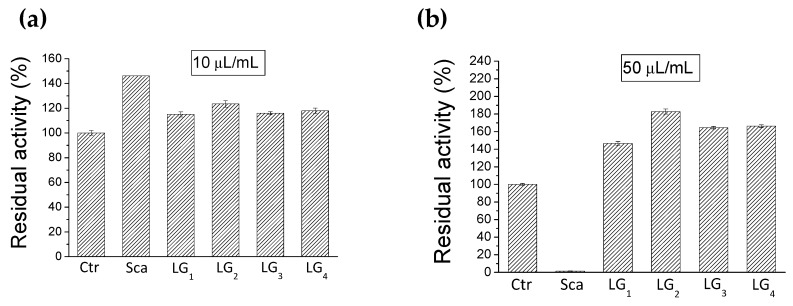
Residual activity of PTP1B enzyme (in vitro %); schaftoside standard (Sca) and Lisosan^®^ G extracts (LG_1_–LG_4_) were tested at two concentrations: (**a**) 10 µL/mL corresponding to 2.7 µM for schaftoside and 1.25 mg/mL for Lisosan^®^ G extracts; (**b**) 50 µL/mL corresponding to 13.5 µM for schaftoside and 6.25 mg/mL for Lisosan^®^ G extracts. Ctrl for control (see experimental section).

The four batches of Lisosan^®^ G and the schaftoside pure standard were in vitro evaluated for their inhibitory effect on PTP1B, known as a negative regulator of insulin receptors. The whole aqueous extracts (LG_1_–LG_4_) at a final concentration of 125 mg/mL and schaftoside, as main flavonoid, at a final concentration of 0.15 mg/mL, were tested adding different amount of extracts (Figure 4). 

**Figure 5:**
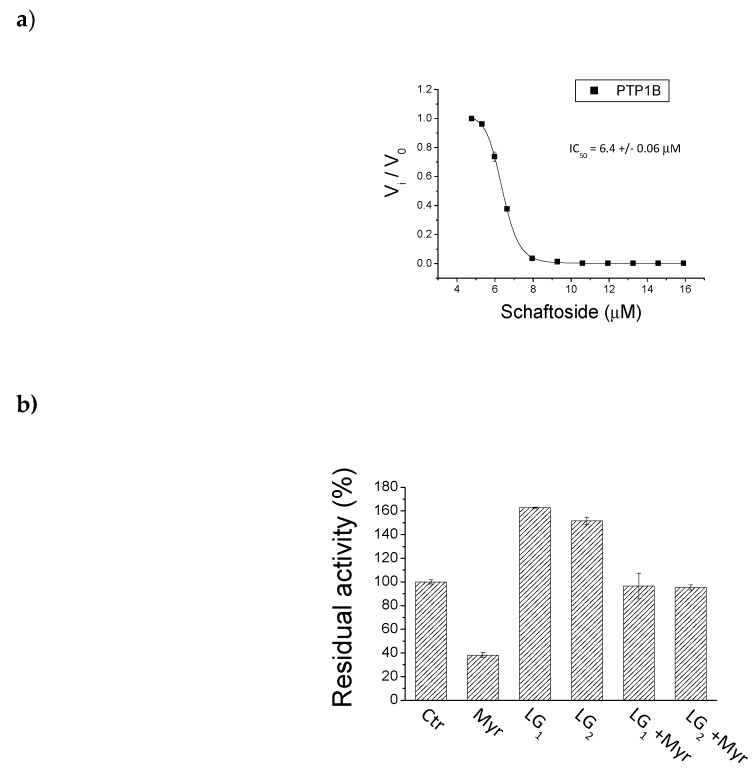
(**a**) IC_50_ of schaftoside. (**b**) Residual activity of PTP1B enzyme (in vitro %) of: Ctrl for control (see experimental section), myricetin standard (Myr), Lisosan^®^ G extracts alone (LG_1_–LG_2_), and added with myricetin (LG_1_+Myr; LG_2_+Myr). The extracts were tested at 10 µL/mL corresponding to 0.5 µM for myricetin and 1.25 mg/mL for Lisosan^®^ G extracts.

The choice of testing schaftoside was also due to the inhibitory activity already showed by flavonoids on PTP1B enzyme as reported by literature [20]. The results pointed out that the whole extracts and the schaftoside were not able to inhibit the enzyme in diluted (10 µL/mL) and in concentrate (50 µL/mL) solutions. Otherwise the pure schaftoside showed a complete inhibition of the enzyme in a concentration of 13.5 µM (Figure 4b) and an IC_50_ value of 6.4 µM (Figure 5a). Considering that the extracts, containing the corresponding amount of schaftoside, resulted not active in the enzymatic assay, we could speculate that other compounds present in Lisosan^®^ G could act as antagonists of schaftoside, presumably hindering its interaction with PTP1B enzyme. This hypothesis was verified by using a myricetin standard added to the LG_1_ and LG_2_ extracts. The choice of myricetin was due to the fact that this flavonol previously showed an IC_50_ of 0.47 µM on PTP1B enzyme (data not shown). Analogously to what observed for the schaftoside, the results in Figure 5b pointed out that the addition of myricetin in the Lisosan^®^ G extracts reduced the inhibitory activity of the molecule alone. In light with these findings, we can conclude that Lisosan^®^ G contains unknown molecules that impede the interaction with the enzyme. These results agrees with recent evidence that confirmed the presence of different allosteric sites on the enzyme surface involved in the regulation of the enzyme activity [21]. Furthermore, it was possible to affirm that the cinnamic components alone were not responsible for such higher inhibitory activity; in fact from literature, ferulic acid in high concentration (100 μM) only exerted a weak inhibition (15%) on PTP1B [22]. Further analyses will be conducted to better define the inhibitory mechanism of schaftoside and the relationship with the applied dose. 

### 2.4. Microbial Composition of Lisosan^®^ G

**Figure 6:**
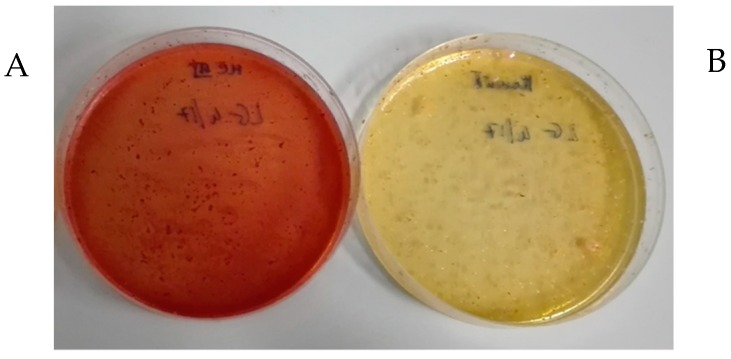
Lisosan^®^ G (LG_1_) was tested for presence of coliforms and staphylococci in (**A**) McConkey III agar medium and (**B**) mannitol salt agar medium, respectively. A total of 500 mg of Lisosan^®^ G was plated. No Colony Forming Unit (CFU) were observed. In (**B**) the medium turned pink to yellow due to the low pH of the fermented product.

Bacteriological analysis firstly excluded the presence of microbial contaminants typical of food specimens, such as coliforms and staphylococci (Figure 6).

Since the microbial composition of Lisosan G is unknown, quantification of specific bacterial species of interest by Real-Time PCR or q-PCR was not applied. *Next-generation sequencing* was chosen as suitable tool to perform the qualitative characterization of bacterial composition. The 16S rRNA gene sequencing was applied to identify the microbial composition of Lisosan^®^ G (Table 3) with *Lactobacillus* (45.4%)*, Enterococcus* (28%), and *Pediococcus* (17%) as the main bacterial genera.

By BLAST alignment we found that the best hits for species identification (99% of identity) were *Lactobacillus fermentum*, *Enterococcus faecium*, and *Pediococcus acidiliacti*. Generally, numerous fermenting bacteria, especially lactic acid bacteria (LAB), have been identified in sourdoughs, including *Lactobacillus* and *Pediococcus* spp. In addition to acidification, the proteolytic activity of LAB allows release of compounds which can promote growth or inhibition and metabolic activities of other microorganisms, as well as organoleptic characteristics. *Enterococcus* genus includes known probiotic strains and was considered a good candidate for co-culture in food fermentation processes [23].

### 2.5. Activity on Lactobacillus reuteri

**Figure 7:**
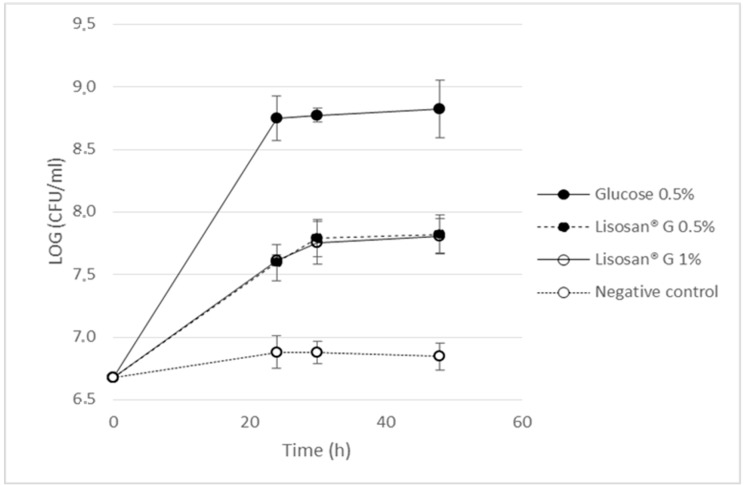
Ability of Lisosan^®^ G (LG_1_) tested at different concentration (0.5 and 1%) in stimulating the growth of *L. reuteri*.

*Lactobacillus reuteri* is a common component of the microbiota of human and animal intestine and it is widely used in probiotic formulations targeted to infants and adults to reduce the incidence and severity of diarrhea, prevent colics and necrotic enterocolitis, and maintain a functional mucosal barrier [24]. As shown in Figure 7, Lisosan^®^ G was capable of stimulating the growth of *L. reuteri* DSM 17938 by more than 1 Log (from 6.7 to 7.8) after 48 h of incubation; even if growth was significantly lower (*p* < 0.05) with respect to that on glucose. The ability to increase the growth of this microorganism is certainly of interest for this fermented matrix characterized by the presence of probiotic strains and phenolic compounds potentially able to exert growth inhibition of harmful bacteria. The reason for the lack of difference in growth potential when Lisosan^®^ G was tested at different concentrations (0.5 and 1%) may also be related to the copresence of different molecules and a broad spectrum of action of this complex product. The beneficial effects of Lisosan^®^ G on human health have already been demonstrated, but prebiotic effects have not been described yet. 

## 3. Materials and Methods

### 3.1. Samples and Reagents

Four batches of Lisosan^®^ G (LG_1_, LG_2_, LG_3_, LG_4_), a food supplement obtained from lysed fine bran and germ of organic wheat grains (*Triticum aestivum*), were provided by Agrisan Srl Company, Larciano (Pistoia, Italy). All solvents used were analytical HPLC grade from Sigma Aldrich (St. Louis, MI, USA). Water was ultrapure (Milli-Q^®^, Merck Millipore, Darmstad, Germany), ferulic acid (purity ≥ 99%), and apigenin (purity ≥ 95%) were purchased from Extrasynthese (Genay, France).

### 3.2. Extraction of Phenolic Compounds

*Free phenols*. Lisosan^®^ G, LG_1_, (250 mg) was extracted with EtOH/H_2_O 80:20 *v*/*v* under magnetic stirring in ultrasonic bath for about 15 min. The extract was centrifuged at 5000 rpm for 10 min. Ten mL of the supernatant were evaporated to dryness and the residue re-dissolved in 1.5 mL of acidified H_2_O (1% HCOOH). The same extraction was performed with H_2_O acidified with 1% HCOOH.

*Bound and total phenols.* An acidic hydrolysis (method A) and three different basic hydrolyses (B_F_, B and B_S_ methods) were applied on the fermented wheat as reported below. All the hydrolyses were carried out by the help of an ultrasonic bath (40 MHz). Method B_F_: 1 g of the sample was extracted with 25 mL of NaOH 0.1 M in MeOH/H_2_O 7:3 *v*/*v*, sonicated for 1 h at 60 °C. The pH was adjusted to neutrality with acetic acid and the solution centrifuged at 5000 rpm for 10 min to recover the supernatant. The same procedure was applied to the solid residue recovered after the extraction of free phenols. Method B was the same of method B_F_ with only a difference on the solvent-dry sample ratio:1 g of Lisosan^®^ G was extracted in 100 mL NaOH 0.1 M in MeOH/H_2_O 7:3 *v*/*v*. Method B_S_: 1 g of the sample was extracted in 25 mL of MeOH/H_2_0 7:3 *v*/*v* with NaOH 4 M, sonicated for 1 h at 60 °C. The pH was adjusted to neutrality with CH_3_COOH and the solution diluted to a final volume of 50 mL with MeOH/H_2_0 7:3 *v*/*v*; the sample was then centrifuged at 5000 rpm for 10 min to recover the supernatant. Method A was an acidic hydrolysis performed on the four batches (LG_1_–LG_4_) using the mixture MeOH/H_2_SO_4_ 9:1 *v*/*v*: 1 g of Lisosan^®^ G was extracted in 25 mL, sonicated for 2 h at 60 °C, and then centrifuged at 5000 rpm for 10 min to recover the supernatant.

### 3.3. Fractionation by Semipreparative HPLC

About 700 mg of Lisosan^®^ G (LG_1_) were almost completely dissolved in 150 mL of HCOOH (1%) under magnetic stirring for 15 min at 60 °C. After centrifugation (5000 rpm, 10 min), the supernatant was dried under vacuum and re-dissolved in 6 mL of distilled water obtaining the total Lisosan^®^ G extract. This extract was fractionated by semipreparative HPLC using a Hewlett Packard 1050 series and a Polaris RP-C18 Ether column (250 × 10 mm, 5 µm, Varian, Germany); 12 fractions were recovered after 30 injections of 100 µL. Elution was carried out at a flow rate of 4 mL min^−1^ with CH_3_CN as solvent A and H_2_O (0.1% HCOOH) as solvent B. A linear elution gradient was employed: solvent A was increased from 0% to 10% in 10 min, from 10% to 15% in 10 min, from 15% to 30% in 10 min, from 30% to 100% in 5 min with a final plateau of 10 min. Total time of analysis was 45 min, equilibration time 10 min. The collection was carried out monitoring the chromatogram at 280 nm up to 15 min, successively 350 nm was the wavelength selected to detect and recover the flavonoids. The fractions were dried, re-dissolved in 1 mL of water, and controlled by analytical HPLC-DAD and MS^n^ analysis.

### 3.4. Inhibition Test on PTP1B Enzyme 

All four batches were treated as follow: the maximum amount of Lisosan^®^ G (250 mg) was dissolved in 50 mL of acidified H_2_O (1% HCOOH), after a magnetic stirring of 15 min. The extracts were then evaporated to dryness and re-dissolved in 2 mL of water. These extracts (LG_1_–LG_4_), the pure schaftoside (at concentration 2.7 µM), and the pure myricetin (at concentration 0.5 µM) were evaluated as inhibitors of the enzyme PTP1B. Furthermore, the IC_50_ was also determined for the schaftoside. Enzymatic assays were carried out using human recombinant PTP1B and *p*-nitrophenylphosphate (pNPP) as reference substrate. According to Paoli et al. [25], the pNPP (2.5 mM final concentration) was dissolved in sodium β,β-dimethyl glutarate buffer (75 mM, pH 7.0), containing 1 mM EDTA and 1 mM dithiothreitol; this solution was used as control (Ctr). Reactions were started by addition of aliquots of the enzyme and stopped by adding 2 mL of KOH 0.2 M. The released p-nitrophenolate was quantified by reading absorbance of the final solution at 400 nm (ε = 18,000 M^−1^ cm^−1^). The net hydrolysis rate was determined subtracting the value of spontaneous hydrolysis rate of pNPP from each sample. The inhibitory power of LG extracts and schaftoside standard was tested adding different amount of extracts (10 and 50 µL/mL) for each assay. Then, the percentage of inhibition was calculated normalizing the absorbance values obtained for assays carried out in the presence of inhibitor versus the control test. All the results were expressed as a mean of three independent experiments. 

### 3.5. Bacteriological Analysis 

In order to exclude the presence of bacterial contaminants of food specimens, such as coliforms and staphylococci, LG_1_ batch was tested in McConkey III medium (Oxoid Basingstone, UK; for the detection of coliform, bacilli, *Salmonella* and *Shigella* species) and in Mannitol salt medium (Oxoid Basingstone, UK; for detection of presumptive pathogenic staphylococci). Lisosan^®^ G (500 mg) were directly plated on the two different media and, after incubation for 24–48 h, presence of potential microbial contaminants were evaluated.

### 3.6. 16S Ribosomal RNA Gene Amplicons Preparation and Illumine MISEQ and Data Analysis

By LG_1_ batch, library of 16S rRNA gene amplicons was prepared by IGA Technology Services (Udine, Italy) through amplification of the V3–V4 hypervariable region. The standard protocol was followed according to the 16S metagenomic sequencing library preparation guide from Illumina (Part #15044223 Rev. B; https://support.illumina.com/, San Diego, CA, USA). Pooled V3–V4 amplicon libraries were sequenced using the Illumina MiSeq platform. Sequence data are available at European Bioinformatics Insitute-EMBL-EBI database [26], under the accession number PRJEB30414. Reads (total number 126.669) were further processed using the MICCA pipeline (version 1.6, http://compmetagen.github.io/micca/, San Diego, CA, USA), as reported by Di Paola et al. [27]. A total of 104.277 Operational Taxonomic Units (OTUs) were assigned by clustering the sequences with a threshold of 97% pair-wise identity. OTU tables for each taxonomic level were created. To deep at species level sequence alignment using Basic Local Alignment Search Tool nucleotide (BLASTn) software (San Diego, CA, USA) in the National Center for Biotechnology Information (NCBI) database was performed. The highest percentage of identity (query cover 100–99% and identity 99%). Expectation value (*E*-value) was used to select significant BLAST hits, keeping only outcomes with the lowest *E*-value (minimal *E*-value of 10^−3^).

### 3.7. Test on Lactobacillus reuteri 

The strain used was obtained from the German Collection of Microorganisms and Cell Cultures (*L. reuteri* DSM 17938). The lyophilized strain was re-vitalized in the Man Rogosa Sharpe (MRS) medium (Oxoid, Basingstone, UK) supplemented with 0.05% cysteine and incubated in anaerobic chamber at 37 °C for 24 h. The MRS medium composition was modified to perform the growth experiment with Lisosan^®^ G in order to eliminate glucose and reduce the concentration of potential growth factors, as described in Khatib et al. [28]. The modified medium is referred to as m-MRS. The prebiotic activity was evaluated using Lisosan^®^ G, LG_1_ batch, at 0.5% and 1% (*w*/*v*) in m-MRS. A positive growth control was performed using m-MRS with 0.5% (*w*/*v*) glucose and a negative control in m-MRS with no added carbon source. The medium containing Lisosan^®^ G as potential carbon source was prepared as follows: the m-MRS ingredients were weighed and the medium autoclaved at 120 °C for 15 min. Lisosan^®^ G was then added, the solution stirred at 80 °C and then autoclaved again at 102 °C for 10 min. The strain was grown overnight in MRS, centrifuged, washed in saline (0.9% NaCl), and re-suspended in saline to obtain an absorbance of 0.7 mAu at 600 nm. This suspension was used to inoculate at 2% (*v*/*v*) the flasks containing the m-MRS medium plus Lisosan^®^ G or glucose or the negative control with no carbon source. The tubes were incubated at 37 °C in anaerobic conditions for 48 h and a 1 mL culture was sampled from each flask, serially diluted, and inoculated on MRS agar plates for viable bacterial counts at pre-established times (0, 24, 30, and 48 h of incubation). Upon incubation, the number of colonies, corresponding to the number of viable cells, were counted and expressed as CFU mL^−1^. The number was transformed into a log10 value (log CFU mL^−1^).

### 3.8. Analytical HPLC-DAD

The extracts from Lisosan^®^ G were centrifuged (5000 rpm, 10 min) and analyzed using a HP 1200L liquid chromatograph equipped with a DAD detector (Agilent Technologies, Palo Alto, CA, USA) using a Poroshell 120, EC-C18 column (150 × 3 mm, 2.7 µm, Agilent, USA). The solvents for the mobile phase were (A) CH_3_CN and (B) 0.1% formic acid/water; the multi-step linear solvent gradient was: 0–5 min 0–10% A; 5–10 min 10–15% A; 10–20 min 15–30% A; 20–25 min 30–35% A; 25–28 min 35–40% A; 28–31 min 40–45% A; 31–42 min 100% A; 42–47 min 100–0% A; equilibration time 5 min; flow rate 0.4 mL min^−1^; injection volume 10 μL. The following wavelengths were simultaneously selected: 240 nm, 280 nm, 330 nm, 350 nm.

### 3.9. MS Analysis of Lisosan^®^ G fractions 

The isolated fractions by semipreparative HPLC were analyzed by direct infusion in ESI-HRMS and MS^n^ on a LTQ-Orbitrap (Thermo Scientific, Bremen, Germany). Each fraction was taken to dryness by evaporation under vacuum and re-suspended in a CH_3_CN/H_2_O mixture, containing 0.1% formic acid. This solution was infused by syringe into the ESI interface of the instrument. Sheath and auxiliary gas flow rates were 10 and 2 (arbitrary units), respectively; capillary voltage and tube lens voltages, as the collision energy and wideband activation voltage in MS^n^ experiments, were optimized for each compound of interest during the infusion. The mass spectrometer was calibrated with the standard mixture indicated by the producer immediately before the acquisition of the samples, both in positive and in negative ion mode.

### 3.10. Quantitative Determination of Phenolic Acids and Flavonoids 

The phenolic acids were evaluated using a five-point calibration curve of ferulic acid at 330 nm, (*R*^2^ = 1, linearity range 0–0.21 µg), while the flavonoid content was determined using a five-point calibration curve of apigenin at 350 nm (*R^2^ =* 0.999, linearity range 0–0.80 µg).

### 3.11. Statistical Analysis 

Each experiment was performed in triplicate, and the results were expressed as the mean values ± SD; the EXCEL software (version 2013, Microsoft Corporation, Redmond, WA, USA) in-house routines were applied. Significance in the prebiotic properties experiment was calculated within each evaluation time with a *t*-test, using the MEANS procedure (SAS). Statistical analysis of data from PTP1B was performed using the Student t-tests, using OriginPro 2018 (OriginLab Corporation, Northampton, MA, 01060, USA http://www.originlab.com) The differences between the groups were considered significant when *p* < 0.05.

## 4. Conclusions

This work improves knowledge on the composition and properties of this fermented grain, and it is the first report to focus on the content of free and bound phenolic compounds in Lisosan^®^ G. Contrary to what reported in literature, the bound phenols remained high (more than 80%), although Lisosan^®^ G was obtained after a fermentation process, usually described as able to increase hydrolytic processes and to strongly reduce the aliquot of bound phenols in cereals. Again, despite some data in the literature, the acidic hydrolysis was able to extract the highest amount of cinnamic derivatives, without degradation of the pool of *C*-glycosylated flavonoids. It was demonstrated that applying both soft basic and acidic hydrolysis on the whole flour it was possible to recover free and bound phenols through only one extractive step with higher yields compared to those obtained with the stronger basic hydrolysis suggested in the literature to date. Regarding the microbial characterization, it showed the presence of bacterial genera with fermentative capability such as *Lactobacillus*, *Pediococcus*, and *Enterococcus,* generally recognized as safe and used in the production of fermented food. The proteolytic activity of these bacteria can contribute to the release of compounds, as phenols, growth of beneficial bacteria as *L. reuteri*, and inhibition of harmful bacteria. For the first time, a prebiotic effect on *L. reuteri* strain, widely used in probiotic formulation targeted to infants, was highlighted. This fermented wheat resulted not able to inhibit the PTP1B enzyme in vitro, otherwise the pure schaftoside, a main *C*-flavonoid of Lisosan^®^ G, showed a strong inhibition power. Schaftoside was tested for the first time on PTP1B enzyme and was active as inhibitor at µM concentration (13.5 µM). Our findings open new perspectives to investigate on the role played by this *C*-glycosylated flavonoid and its analogous, typically present in wheat and in other cereals. Further studies are desirable to clarify the mechanism linked to the action of schaftoside and to test also other similar *C*-flavonoids on PTP1B enzyme. The International Diabetes Federation announced that the global population of diabetics in 2015 was close to 400 million, and this number could rise to 600 million in 2040. In this context, research on new functional foods that can help stem the onset of this disease in the near future is recognized as strategic. Overall, our results provide further insights on the nutraceutical potential of this fermented food, whose beneficial effects were previously demonstrated by recent in vivo and ex vivo experiments.

## Figures and Tables

**Table 1:** *C*-glycosylated flavonoids detected in Lisosan^®^ G by MS^n^ experiments.

		Positive Ion MS^n^		Negative Ion MS^n^	
Analytes	C-glycosylated Flavonoids	[M − H]^+^	Fragment Ions	[M − H]^−^	Fragment Ions
**9**	Carlinoside/Neocarlinoside/Isocarlinoside	581	563; 545; 527; 509	579	489; 399
**10**	Carlinoside/Neocarlinoside/Isocarlinoside	581	563; 545; 527; 509	579	489; 399
**11**	Isoschaftoside	565	547; 529; 511; 427; 349	563	473; 353; 325
**13**	Schaftoside	565	547; 529; 511; 427; 349	563	473; 353; 325

**Table 2:** Concentration of the phenolic compounds identified in Lisosan^®^ G applying different extraction methods and evaluated through HPLC-DAD by suitable external standards. (**a**) free phenols (**FP**), and total phenols determined after basic hydrolyses (methods **B_S_**, **B_F_**, **B**) applied to Lisosan^®^ G first batch (LG_1_); (**b**) total phenols determined after acidic hydrolysis (method **A**) applied to the four batches of Lisosan^®^ G (**LG_1_**; **LG_2_**; **LG_3_**; **LG_4_**). The data are a mean of three independent extractions expressed as mg/100 g dry product. The relative standard deviation (RSD) was below 4% for all the detected phenols.

**(a) Free (FP) and Total Phenols (B_s_, B_f_ and B) from Basic Hydrolyses in mg/100 g**				
**Compounds**	**FP**	**B_s_**	**B_F_** (on whole flour)	**B**
**Carlinoside/isocarlinoside/neocarlinoside (9)**	6	-	4	2
**Carlinoside/isocarlinoside/neocarlinoside (10)**	5	-	3	2
**Isoschaftoside (11)**	7	6	7	13
**Schaftoside (13)**	17	12	15	22
**Ferulic Acid (15)**	3	223	12	48
**Methyl Ferulate (19)**	-	-	70	178
**Total ferulates**	3	223	82	226
**Total phenols**	38	241	111	265
**(b) Total Phenols Obtained Applying the Acidic Hydrolysis mg/100g**				
**Compounds**	**LG_1_**	**LG_2_**	**LG_3_**	**LG_4_**
**Carlinoside/isocarlinoside/neocarlinoside (9)**	2	2	2	2
**Carlinoside/isocarlinoside/neocarlinoside (10)**	3	2	3	3
**Isoschaftoside (11)**	10	6	6	6
**Schaftoside (13)**	17	10	9	10
**Ferulic Acid (15)**	2	2	3	2
**Methyl Ferulate (19)**	245	197	208	158
**Total ferulates**	247	199	211	160
**Total phenols**	279	219	231	181

**Table 3:** Microbial composition of Lisosan^®^ G (LG_1_). Number of sequenced reads and amount (%) of identified taxa were reported.

					N. Reads	Amount (%)
Phylum	Class	Order	Family	Genus		
**Firmicutes**	Bacilli	Lactobacillales	*Lactobacillaceae*	*Lactobacillus*	47,344	45.4%
**Firmicutes**	Bacilli	Lactobacillales	*Enterococcaceae*	*Enterococcus*	29,195	28.0%
**Firmicutes**	Bacilli	Lactobacillales	*Lactobacillaceae*	*Pediococcus*	17,711	17.0%
**Firmicutes**	Bacilli	Lactobacillales	Unclassified	Unclassified	3863	3.7%
**Firmicutes**	Bacilli	Lactobacillales	*Lactobacillaceae*	Unclassified	3391	3.3%
**Firmicutes**	Bacilli	Lactobacillales	*Streptococcaceae*	*Lactococcus*	1218	1.2%
**Unclassified**		Unclassified	Unclassified	Unclassified	918	0.9%
				Others	637	0.6%
				Total	104,277	

## References

[1] Terefe N.S. (2016). Reference Module In Food Science. Food Fermentation.

[2] Hur S.J., Lee S.Y., Kim Y.C., Choi I., Kim G.B. (2014). Effect of Fermentation on the Antioxidant Activity in Plant-Based Foods. Food Chem..

[3] Dey T.B., Kuhad R.C. (2014). Enhanced Production and Extraction of Phenolic Compounds from Wheat by Solid-State Fermentation with Rhizopus Oryzae RCK2012. Biotechnol. Rep..

[4] Arranz S., Saura Calixto F. (2010). Analysis of Polyphenols in Cereals May Be Improved Performing Acidic Hydrolysis: A Study in Wheat Flour and Wheat Bran and Cereals of the Diet. J. Cereal Sci..

[5] Holscher H.D. (2017). Dietary Fiber and Prebiotics and the Gastrointestinal Microbiota. Gut Microbes.

[6] Longo V., Gervasi P.G., Lubrano V. (2011). Cisplatin Induced Toxicity in Rat Tissues: The Protective Effect of Lisosan G. Food Chem. Toxicol..

[7] Lubrano V., Baldi S., Napoli D., Longo V. (2012). Beneficial Effect of Lisosan G on Cultured Human Microvascular Endothelial Cells Exposed to Oxidised Low Density Lipoprotein. Indian J. Med. Res..

[8] Lucchesi D., Russo R., Gabriele M., Longo V., Del Prato S., Penno G., Pucci L. (2014). Grain and Bean Lysates Improve Function of Endothelial Progenitor Cells from Human Peripheral Blood: Involvement of the Endogenous Antioxidant Defenses. PLoS ONE.

[9] La Marca M., Beffy P., Pugliese A., Longo V. (2013). Fermented Wheat Powder Induces the Antioxidant and Detoxifying System in Primary Rat Hepatocytes. PLoS ONE.

[10] Verma M., Gupta S.J., Chaudhary A., Garg V.K. (2017). Protein Tyrosine Phosphatase 1B Inhibitors as Antidiabetic Agents—A Brief Review. Bioorg. Chem..

[11] Dinelli G., Segura-Carretero A., Di Silvestro R., Marotti I., Arráez-Román D., Benedettelli S., Ghiselli L., Fernadez-Gutierrez A. (2011). Profiles of Phenolic Compounds in Modern and Old Common Wheat Varieties Determined by Liquid Chromatography Coupled with Time-of-Flight Mass Spectrometry. J. Chromatogr. A.

[12] Leoncini E., Prata C., Malaguti M., Marotti I., Segura-Carretero A., Catizone P., Dinelli G., Hrelia S. (2012). Phytochemical Profile and Nutraceutical Value of Old and Modern Common Wheat Cultivars. PLoS ONE.

[13] Rahman M.A., Akond M., Babar M.A., Beecher C., Erickson J., Thomason K., De Jong F.A., Mason R.E. (2017). LC-HRMS Based Non-Targeted Metabolomic Profiling of Wheat under Post-Anthesis Drought Stress. Am. J. Plant Sci..

[14] Iswaldi I., Arráez-Román D., Rodríguez-Medina I., Beltrán-Debón R., Joven J., Segura-Carretero A., Fernández-Gutiérrez A. (2011). Identification of Phenolic Compounds in Aqueous and Ethanolic Rooibos Extracts (Aspalathus Linearis) by HPLC-ESI-MS (TOF/IT). Anal. Bioanal. Chem..

[15] Colombo R., Yariwake J.H., Mccullagh M. (2008). Study of C- and O-Glycosylflavones in Sugarcane Extracts Using Liquid Chromatography—Exact Mass Measuremente Mass Spectrometry. J. Braz. Chem. Soc..

[16] Simirgiotis M.J., Schmeda-Hirschmann G., Bórquez J., Kennelly E.J. (2013). The Passiflora Tripartita (Banana Passion) Fruit: A Source of Bioactive Flavonoid C-Glycosides Isolated by HSCCC and Characterized by HPLC-DAD-ESI/MS/MS. Molecules.

[17] Brandolini A., Castoldi P., Plizzari L., Hidalgo A. (2013). Phenolic Acids Composition, Total Polyphenols Content and Antioxidant Activity of Triticum Monococcum, Triticum Turgidum and Triticum Aestivum: A Two-Years Evaluation. J. Cereal Sci..

[18] Adom K.K., Liu R.H. (2002). Antioxidant Activity of Grains. J. Agric. Food Chem..

[19] Tanaka A., Kato A., Tsuchiya T. (1964). Isolation of β-Sitosteryl Ferulate from Rice Bran Oil. J. Jpn. Oil Chem. Soc..

[20] Jiang C.S., Liang L.F., Guo Y.W. (2012). Natural Products Possessing Protein Tyrosine Phosphatase 1B (PTP1B) Inhibitory Activity Found in the Last Decades. Acta Pharmacol. Sin..

[21] Hjortness M.K., Riccardi L., Hongdusit A., Zwart P.H., Sankaran B., De Vivo M., Fox J.M. (2018). Evolutionarily Conserved Allosteric Communication in Protein Tyrosine Phosphatases. Biochemistry.

[22] Adisakwattana S., Pongsuwan J., Wungcharoen C., Yibchok-Anun S. (2013). In Vitro Effects of Cinnamic Acid Derivatives on Protein Tyrosine Phosphatase 1B. J. Enzym. Inhib. Med. Chem..

[23] De Vuyst L., Leroy F., Foulquie M.R. (2003). Enterococcus Faecium RZS C5, an Interesting Bacteriocin Producer to Be Used as a Co-Culture in Food Fermentation. Int. J. Food Microbiol..

[24] Urbańska M., Szajewska H. (2014). The Efficacy of Lactobacillus reuteri DSM 17938 in Infants and Children: A Review of the Current Evidence. Eur. J. Pediatr..

[25] Paoli P., Cirri P., Caselli A., Ranaldi F., Bruschi G., Santi A., Camici G. (2013). The Insulin-Mimetic Effect of Morin: A Promising Molecule in Diabetes Treatment. Biochim. Biophys. Acta Gener. Subj..

[26] Microbial Composition of a Fermented Whole Wheat (Triticum aestivum). http://www.ebi.ac.uk/ena/data/view/PRJEB30414.

[27] Di Paola M., Bonech E., Provensi G., Costa A., Clarke G., Ballerini C., Filippo C.D., Passani M.B. (2018). Oleoylethanolamide Treatment Affects Gut Microbiota Composition and the Expression of Intestinal Cytokines in Peyer’s Patches of Mice. Sci. Rep..

[28] Khatib M., Giuliani C., Rossi F., Adessi A., Al-Tamimi A., Mazzola G., Di Gioia D., Innocenti M., Mulinacci N. (2017). Polysaccharides from By-Products of the Wonderful and Laffan Pomegranate Varieties: New Insight into Extraction and Characterization. Food Chem..

